# Synthesis and Characterization of Wood Rigid Polyurethane Composites

**DOI:** 10.3390/ma15124316

**Published:** 2022-06-18

**Authors:** Hamza Bradai, Ahmed Koubaa, Hassine Bouafif, Armand Langlois, Basma Samet

**Affiliations:** 1 Institut de Recherche sur Les Forêts, Université du Québec en Abitibi-Témiscamingue, 445 Boul. Université, Rouyn-Noranda, QC J9X 5E4, Canada; hamza.bradai@uqat.ca; 2 Centre Technologique des Résidus Industriels (CTRI), Rouyn-Noranda, QC J9X 5E5, Canada; hassine.bouafif@cegepat.qc.ca; 3 EnerLab, 1895 Chemin de l’Industrie, Saint-Mathieu-de-Beloeil, QC J3G 4S5, Canada; alanglois@enerlab.ca; 4 National Engineering School of Sfax (ENIS), University of Sfax, Sfax 3038, Tunisia; basma.samet@enis.tn

**Keywords:** wood polyurethane composite, rigid polyurethane foam (RPUF), wood chips, mechanical properties, thermal conductivity, fire retardancy

## Abstract

Incorporating biodegradable reinforcement, such as wood particles, into rigid polyurethane foams (RPUFs) is among the alternatives to reduce their environmental impact. This study aims to assess the effect of different wood particles as reinforcement in RPUFs. Reinforced rigid polyurethane foams are synthesized with milled wood particles of various forms and sizes and commercial polyol and isocyanate. The effect of fiber treatments and mechanical stirring on foams’ properties is also studied. Additional tests on polyisocyanurate foams (PIR) were undertaken to assess the effect of reinforcement on their properties. Mechanical properties are measured to investigate the impact of wood particle reinforcement on the foam. Confocal microscopy and Fourier-transform infrared spectroscopy (FTIR) showed the interaction between the wood fibers and the matrix. Despite the adhesion observed for some fibers, most of the cell walls of RPUFs were punctured by the rigid wood fibers, which explained the decrease in the compressive strength of the composites for manually mixed foams. Mechanical stirring proved to be an efficient method to enhance the reinforcement power of untreated fibers. RPUF foams’ properties showed similar changes when untreated wood flour was introduced to the formula, increasing compressive strength significantly.

## 1. Introduction

Wood is one of Canada’s most important natural resources. For example, in Quebec, forests cover nearly 761,100 km^2^, which represents almost half of the territory [1]. The exploitation of this resource creates an enormous amount of waste. Wood chips are an example of this, with an almost constant production between 2010 and 2017 of approximately 4.55 million dry metric tons (dmt). On the other hand, sawdust increased from 1.19 million dmt in 2010 to 1.54 million dmt in 2017 [1]. There are specific valorization routes for each category of these residues, mainly for bioenergy applications. As for wood chips, they are generally used for pulp and paper production. However, needle-shaped shavings and sawdust are under-exploited because of their bad wettability and negative effect on the pulp yield and paper properties [2]. Furthermore, the paper industry is experiencing a crisis reflected by the decrease in pulp, paper, and cardboard producers, from 63 in 2005 to 41 in 2017 [1]. This translates into reducing the use of wood chips by these industries from 6.3 million dmt in 2007 to 4.83 million dmt in 2017. Between 2008 and 2017, pulp production decreased by 32%, and paper and paperboard decreased by 34.4% [1]. The important decrease in the demand for newsprint paper and the use of recycled paper explain this reduction. Therefore, valorization of wood particles and residues such as needle-shaped shavings and sawdust as reinforcement in composites is becoming increasingly attractive. One avenue for valorization is rigid polyurethane foams (RPUF) because of the chemical similarity of wood to the polyols used in RPUFs. The hydroxyl groups in wood represent a nucleation site for the reaction with the isocyanate, which is the basic chemical reaction for RPUF foams production.

Remaining constant since 2010, the production of wood chips is becoming increasingly problematic for sawmills that can no longer find buyers, thus decreasing the price and revenue. For example, Quebec’s wood chips revenues decreased by 55% between 2008 and 2017 [1]. Hence, finding new markets for valorizing wood chips is very important. For example, they can be valorized in the construction sector as a reinforcement for polyurethane insulation materials thanks to their thermal and acoustic properties [3,4,5]. Polyurethane is also used in construction as an adhesive for aesthetic and structural applications [6]. However, given their origin from fossil oil, an alternative of bio-sourced products will be useful and appreciated by the market [7]. As a result, there is a growing trend toward incorporating natural fibers in polymeric matrices, especially wood chips. Indeed, previous studies have proven that their use can benefit from an economic and ecological point of view [8,9]. Using cellulose nanocrystals to reinforce palm oil polyurethane foams increased compressive strength and led to superior dimensional stability [7]. Using wheat straw lignin as reinforcement for rigid polyurethane enhanced thermal insulation and maximum compressive strength [10]. Lignin was also proven to be an adequate substitute for polyol [11]. Saffar et al. [12] reported that a 20% substitution of the polyol with oxypropylated lignin resulted in an increase of 17% in the modulus of elasticity and 5.5% in insulation properties [12].

This project incorporated wood chips into non-recyclable foams to reduce their environmental impact. The major challenge for bio-based products is introducing high proportions of bio-sourced materials. Reinforcing foams with lignocellulosic fibers could help achieve this goal.

In this study, a commercial polyurethane formulation and wood fibers from milled wood chips of different sizes served to manufacture polyurethane (RPUF) biocomposites using a mechanical and a manual process. We also investigated the reinforcement capacity of the fibers on a polyisocyanurate foam and the impact of wood chips’ acetylation on the processing and properties of the RPUF biocomposites. The use of wood particles for polyurethane composites is among the solutions for the valorization of wood residues and by-products for more lucrative bioproducts than bioenergy applications. Fiber with important variations in morphology and chemical structures also contributes to understanding the interfacial adhesion between the wood fiber and polyurethane matrix.

## 2. Materials and Methods

### 2.1. Materials

The wood chips used in this study were provided by the research center on renewable materials at Laval University. Black spruce (*Picea mariana* Mill.) chips were received as sawdust and wood fines with various particle sizes. The wood incorporated in the formulation of RPUF foams has been ground to more homogeneous particle sizes; fine (<0.106 mm), medium (0.1–0.3 mm), and coarse (0.3–0.5 mm). Three types of fibers with different treatments were also used: kraft chemical pulp fibers, microcrystalline cellulose (MCC) under the commercial name Hewetten 102, both provided by J. Retenmaier (Fosston, MN, USA), and black spruce wood fibers treated in the laboratory by acetylation with acetic acid and hydrogen peroxide. ENERLAB 2000 Inc. (Saint-Mathieu-de-Beloeil, QC, Canada) supplied the resin (Enerthane Resin 1142), consisting of a mixture of polyols, blowing agent (Hydrofluorocarbons), dimethylclohexylamine catalyst, and a fire retardant. The second polyol was used to produce polyisocyanurate (PIR) foams. The used isocyanate consists of a mixture of diphenylmethane diisocyanate, isomers, homologs, and 4,4′-diphenylmethane diisocyanate (4,4′-MDI). Table 1 summarizes the composition of the RPUF foam used.

### 2.2. Wood Preparation

The chips in different sizes were shredded with a Retsch shredder with a 4 mm, 2 mm, and 1 mm grid. The crushed product was screened into the following classes: fine (<0.106 mm), medium (0.1–0.3 mm), and coarse (0.3–0.5 mm). Before adding the fibers to the foam formulation, all fibers were oven-dried at 60 °C for 24 h to reduce and stabilize their moisture content (3–7%).

### 2.3. Preparation of Reinforced RPUFs

The preparation of the mixtures to produce the foam was carried out in two parts. It is a mixture between components A (Enerthane resin 1142) and B (isocyanate) in a cardboard glass. For wood fiber reinforced foams, the first part includes the mixture of polyols and different additives with reinforcement. The different reinforcement percentages represent the mass percentage of polyol (parts per hundred polyols (php)). The polyol was weighed and poured first, and the calculated amount of reinforcement was gradually added while mixing manually.

The mixing time was set at 5 min, which produces a visibly homogeneous mixture. The final step was to add the amount of isocyanate with a mass ratio of 1:1 to the polyol. The mix of polyol/fiber and isocyanate was mixed for 10 to 15 s, depending on the reactivity of different formulations. The mixing time between polyol and isocyanate was set at 10 s. This is the time it takes for the mixture to become more viscous and the emulsion to start.

The method used in this study was the “free-rising one shot method”. It involves mixing all additives in the polyol, and then the isocyanate is added at once. The foams swell without external heat because of the exothermic nature of the reaction. The foams were then cured at room temperature for 72 h in the cardboard glasses. Once cured, the foams were cut into 5 × 5 × 5 cm cubes for density and compression tests. Small thin samples were also cut for microscopy tests. The samples were conditioned for 48 h according to ASTM D1621 before characterization.

### 2.4. Process Optimization

#### 2.4.1. Mechanical Mixing Process

A Caframo stirrer BDC3030 mixed the polyol, the additive, and the fiber mixture at a constant speed of 150 rpm and a residence time of 10 min. The time and speed were taken from a previous study that optimized the mixing parameters and investigated their effect on the properties of reinforced RPUF foams [13]. The rest of the foam preparation steps were the same as the manual process. This consisted of putting the mixture (polyol, additive, and reinforcement) into a cardboard glass to which isocyanate was added in one go. The resulting reaction medium was then mixed for 10 to 15 s. After the foam had swollen, it was kept at room temperature for the maturation phase for 48 h before being cut. Compression test samples were cut in the shape of a cylinder 7 cm in diameter in the center of the foams.

#### 2.4.2. Fibers Treatment

Wood flour, obtained by grinding black spruce chips supplied by the Centre for Research on Renewable Materials at Laval University, with fine grain size, was treated to modify its chemical structure. The goal was to improve its adhesion with the matrix. An acetylation treatment was applied to the wood fibers. The treatment consisted of immersing the reinforcement in glacial acetic acid, hydrogen peroxide, and water in a ratio of 50:38:12 at 60 °C with continuous agitation [14]. The fibers were then neutralized with distilled water and dried. Fibers were dried under a hot airflow to avoid the formation of aggregates. Kraft fibers and microcrystalline cellulose (MCC) were also used to evaluate their effect on the properties of the RPUF foams.

### 2.5. Polyisocyanurate Foams

Polyisocyanurate-based foams were produced after considering the statistical analysis of preliminary results to optimize the polyurethane foam formulations (Table S1). The statistical analysis is presented in the Supplementary Materials. The preparation of PIR foams was the same as RPUF with a 5-s mixing time because of isocyanate reactivity. In this analysis, a composite experimental plan determined the optimal formulations in density and mechanical resistance using the NEMROD statistical analysis [15]. The iso-response surfaces of density and mechanical properties gave a simple visualization of the optimum fiber size and proportion (Figure S1). The NEMROD statistical analysis identified foam formulations’ optimum percentage and fiber size. 

### 2.6. Nomenclature

Table 2 presents the foam formulation. The nomenclature PU_XY will designate the prepared foams for polyurethane foams and PI_XY for PIR foams such that X and Y represent the nature and percentage of reinforcement, respectively. The nature X denotes the particle size for untreated fibers and the type of treatment for treated fibers. Table 3 gives these different meanings. Y indicates the percentages of fibers used—10, 20, and 30% wood fibers in part per hundred grams of polyol (php). Foams without reinforcement will be denoted PU0 and PIR0. Foams with mechanical mixing will have the nomenclature PUM_XY.

## 3. RPUF’s Characterization

### 3.1. Apparent Density Measurement

The foams obtained were cut according to ASTM D 1622 and were conditioned at 23 °C and 50% relative humidity for 48 h in a conditioning chamber. Sample weights and dimensions were measured to the nearest 0.01 g and 0.02 mm, respectively, using five replicates per formulation. The weight to volume ratio gave the apparent density.

### 3.2. Compressive Strength Evaluation

**Figure 1:**
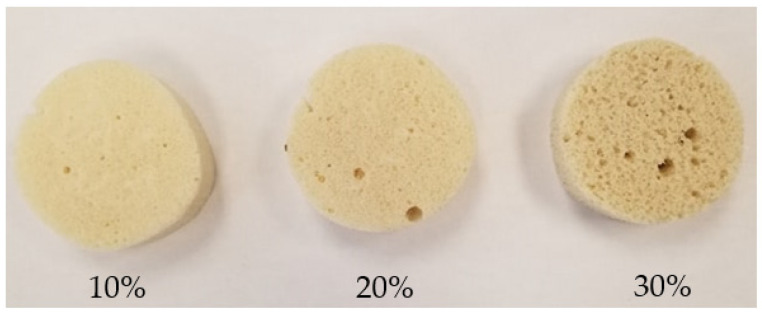
Numerical image of mechanically mixed foams with fine fiber at 10, 20, and 30%.

The samples were cut into cubes (50 × 50 × 50 mm) for the manually mixed formulations and cylinders of 7 cm diameter for the rest of the samples (Figure 1). According to the ASTM D1621, all tests were performed using the Zwick Z020 machine (Zwick Rowell, Germany) at a compression speed of 10% of the sample thickness per minute. Five repetitions were carried out for each test. The test stopped after reaching at least 15% deformation. Compressive strength was calculated at 10% deformation or the point of deflection.

### 3.3. Confocal Microscopy

The confocal laser microscope (Keyence, VK-X150 100) characterized the cell morphology to understand the effect of adding the wood reinforcement and its particle size on the different foams. Indeed, using this microscope, visualization was not performed directly. The images obtained are a two-dimensional recomposition taken at different depths and reconstructed by computer. This avoids the problem of the fine cuts required by the optical microscope. This test makes it possible to understand the interaction between the reinforcement and the polyurethane matrix by analyzing the size and shape of the foam cells.

In addition, the wood fiber wall was analyzed along with its interaction and location in the different cells of the foam. A toluidine blue stain was used, allowing better visibility of the fibers in the foam. A low-concentration solution was prepared in which the samples to be analyzed were immersed for 15 min. Before observing under the microscope, the samples were washed with distilled water and dried.

### 3.4. Thermogravimetric Analysis

Thermogravimetric analysis (TGA) provides information on the material’s degradation process as a temperature function. This makes it possible to evaluate the temperatures at which mass loss occurs. The TGA tests were carried out on different foam formulations to assess the influence of the wood reinforcement on the start and end temperatures of the degradation of RPUF and PIR foams. The device used in this study was the TA instrument model Q50 with a temperature sweep of 25 to 600 °C and a heating rate of 20 °C/min under a nitrogen atmosphere. The TGA and DTG curves were analyzed, representing the mass loss and the derivative of mass loss versus temperature.

### 3.5. Cone Calorimeter Test

For this test, wood molds with a Plexiglas coating were used to obtain foams with dimensions (100 × 100 × 50 mm). The foams were mixed in the cardboard cups and poured into the molds. After a curing period of 72 h, the foams were removed from the molds and cut into lamellas with dimensions (100 × 100 × 8 mm). All samples were polished to ensure a constant thickness. The test configurations used in this study simulated a widely developed fire in a closed environment with a flow of 50 kW/m^2^. Foams’ fire retardancy properties measured by this test were: Total heat release (THR), Total oxygen consumption (TOC), Total smoke released (TSR), Mean CO yield (MCOY), Mean CO_2_ yield (MCO_2_Y), and maximum average rate of heat emission (MARHE). The test was conducted according to the ASTM E 1357 standard with two duplicates for each sample.

### 3.6. Thermal Conductivity Test

The primary use for polyurethane foam is in insulation. It is important to quantify its thermal conductivity. This test involves placing the sample between two plates with different surface temperatures. The test was performed according to ASTM E1530 with a DTC 300 TA Instrument. The samples used were discs of 50 mm in diameter and 15 mm in height. The thermal conductivity of each sample was calculated and reported.

## 4. Results and Discussion

### 4.1. Effect of Wood Flour on RPUFs Properties

#### 4.1.1. Density

**Figure 2:**
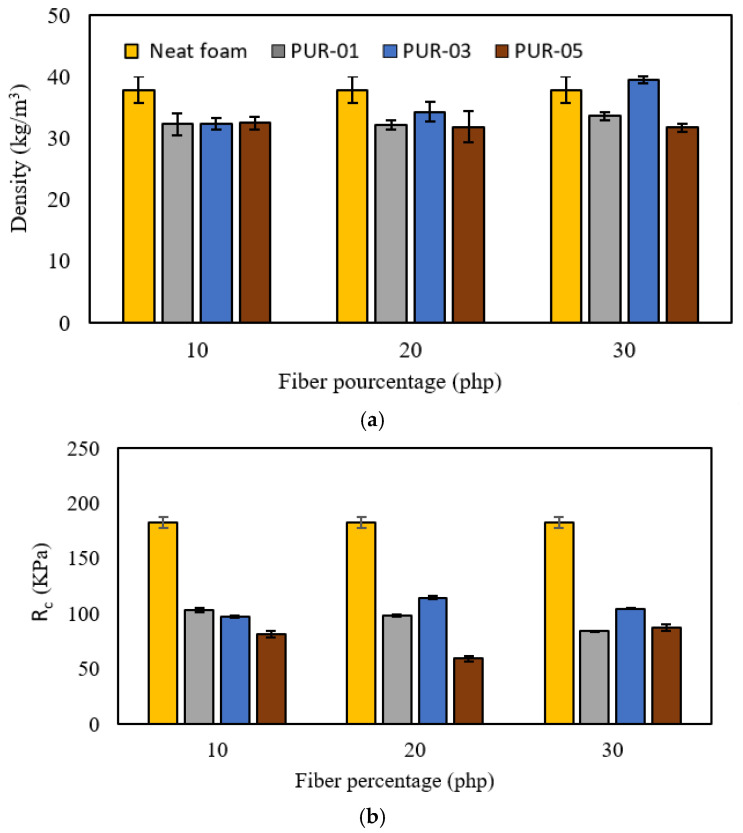
Effect of wood particles size and proportion on reinforced polyurethane foams properties; (**a**) density and (**b**) Compressive strength.

The variation in the density of the foams at different percentages and particle sizes was studied and shown in histogram form in Figure 2. The results showed a slight variation in density between unreinforced (reference) and wood-reinforced foams. The general trend is a slight decrease in the latter.

Compared to foam without reinforcement, the decrease in density for PUR_01 and PUR_05 foams could be explained because wood disturbs the main reaction between polyol and isocyanate. This is in line with other research, which found that wood reinforcement with a fine grain size acts as a nucleation site in the foam. This creates more cells and subsequently more microvoids, thus reducing the properties of the resulting foams, such as density [16]. The specific surface area is very high for the finest particle size, which could induce a higher hydroxyl number, thus reducing the polyol and isocyanate interactions.

The increase in density for PUR_03 foams (medium particle size) could mean that this particle size has a specific surface area that allows the fibers to contribute to the reactions with the isocyanate without interfering with the crosslinking reactions (polyol-isocyanate). Variations may also correlate with the interaction of the fibers with the isocyanate by reducing the swelling rate contributing to the increase in density.

Yuan and Shi (2009) demonstrated that the hydroxyl number of the polyol affects the crosslinking reactions in polyurethane foam [17]. A higher index leads to more crosslinking since the amount of isocyanate is insufficient to react simultaneously with the polyol and wood. The reaction of the isocyanate with the wood could simulate using a polyol with fewer hydroxyl bonds because some of the hydroxyl groups will not react because of the decrease in free isocyanate. In addition, fiber residues were present in the lower part of the samples. The higher density of the chips compared to the foam explains this result.

#### 4.1.2. Mechanical Properties

Figure 2 shows a decrease in compressive strengths for all formulations. In other research, Chang (2014) has shown similar results by adding 10% of untreated wood fibers. The data collected show that, depending on the particle size, the effect of percentage on the compressive strength is different [13].

For PUR_01 foams, the compressive strength (Rc) decreases as the percentage of reinforcement increases. Indeed, Rc goes from 103 kPa for PUR_01-10 to 83 kPa for PUR_01-30. Considering the specific higher surface area of smaller fibers and the increase in the viscosity of the mixture, we can assume that the main reactions between isocyanate and polyol are not occurring properly.

Reinforcement with average grain size (0.1–0.3 mm) shows different behavior when the reinforcement’s proportion increases. This grain size shows the best resistance for a percentage of 20%, which reaches almost 120 kPa. This represents the highest compressive strength obtained for the different formulations. However, this resistance decreases for PUR_03-30 to reach 104 kPa. Once more, the biggest problem here is the mixture’s viscosity at 30% reinforcement which could explain the decrease in compressive strength. This increase in viscosity has an even more negative effect as the mixing time between isocyanate and polyol is about 15 s. Then, gas bubbles appear, and the foam starts to swell. This step is critical to the integrity of the foam’s three-dimensional structure and interfering with this phase reduces the properties of the foams. Extending the mixing time could contribute to the collapsing of the foam.

**Figure 3:**
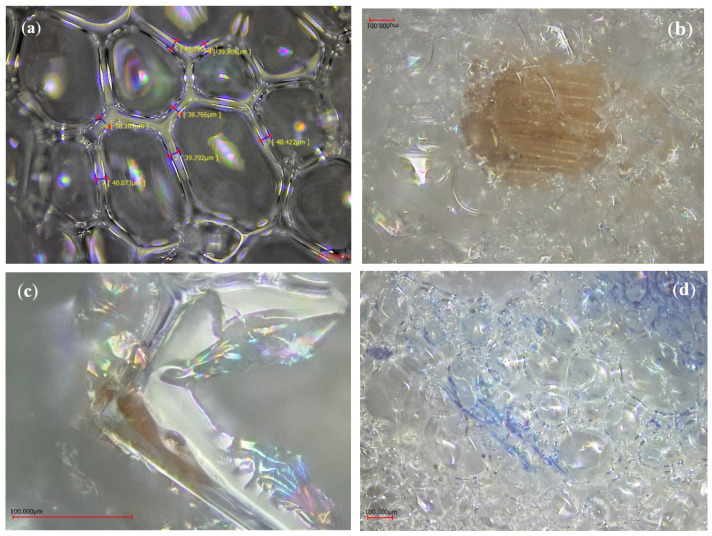
Cell morphology and fiber/matrix interaction: (**a**) Neat foam, (**b**) PUR-05, (**c**,**d**) PUR-01.

Foams with coarse-grained reinforcements showed the lowest compressive strengths and followed a different behavior. Indeed, the Rc decreases from PUR_05-10 to PUR_05-20 but increases for foam with 30% reinforcement. This could be explained by the fact that these fibers cannot be incorporated into the cell walls resulting in anisotropic cells, thus reducing the compressive strength of the foam [18]. Figure 3 shows the effect of adding wood fibers on cell structure. Figure 3a shows the foam without reinforcement with homogenous cells. The addition of coarse fibers (Figure 3b) shows that these fibers cannot be encapsulated in the cell wall, compared to smaller reinforcement (Figure 3c,d), where the fiber can be trapped in the cell wall. Still, the overall cell structure is much more heterogeneous. This is explained by the fact that the fibers act as nucleation points to create more cells, hindering the foam’s homogenous cell growth.

#### 4.1.3. Thermal Stability

**Figure 4:**
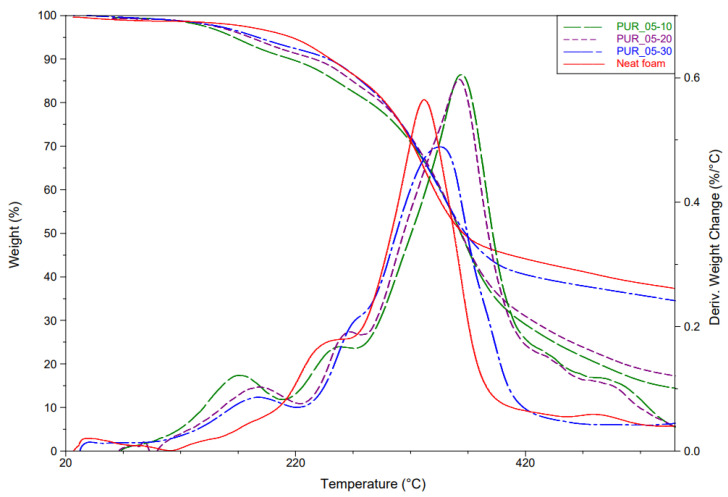
Effect of the wood flour reinforcement on TG and DTG of the reinforced polyurethane foams.

Figure 4 shows the mass loss and its derivative curves as a function of temperature for foams prepared with coarse-grained particles (0.3–0.5 mm) at different percentages. The literature states that the degradation of polyurethane foams made with MDI isocyanate begins between 220 °C and 270 °C, representing the unstable urethane degradation [19,20,21]. The degradation temperature of foams depends on the nature of the isocyanate, and the polyol used [18]. Other research has found that MDI and polyether-based polyurethane degradation occurs at 350 °C [22]. In this study for non-reinforced foam, two degradation temperatures were observed, the first at 235 °C and the second at 340 °C, corresponding to the two-stage degradation of polyurethane foams. The first decrease in mass at 235 °C is related to the degradation of urethanes, and the second corresponds to the degradation of the polyol backbone used.

An additional degradation point between 150–200 °C is present in the curves with wood reinforcements for the different percentages. Indeed, Orfao et al. (1999) have shown that the degradation temperature of cellulose starts around 225 °C and hemicellulose at about 160 °C [23]. This would explain the degradation peak between 150 and 200 °C in the curves of wood-reinforced foams. All the foams produced in this study show the same thermal behavior regardless of the size or percentage of wood added to the foam.

#### 4.1.4. Fire Retardancy

**Figure 5:**
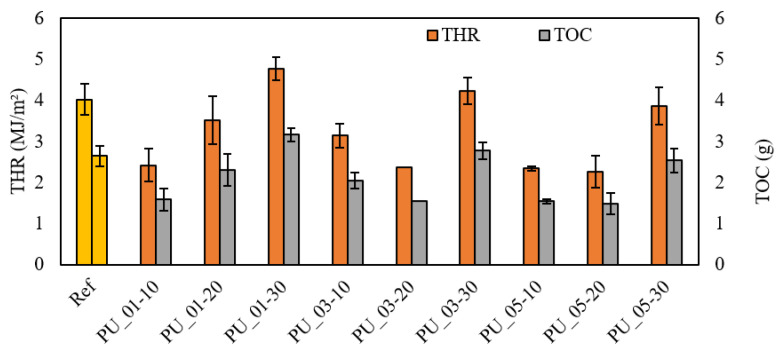
Effect of particle size and percentage on the total heat release and total oxygen consumption.

The fire retardancy properties of the reinforced foams are compared to those of the non-reinforced foam. Several parameters can be derived from the cone calorimeter tests, but this section focuses on the safety aspect of using these foams in the building industry. The THR, TOC, MCOY, MCO_2_Y, TSR, and MARHE values described above will be studied to evaluate the effect of the addition of wood reinforcement on RPUF foams’ fire retardancy [24,25]. These factors are important in a real fire situation because they inform us about the total flow with which the material contributes to the development of the fire (THR) and the amount of oxygen consumed by the flames (TOC). Figure 5 shows the results for THR and TOC, which follow the same trend with a value that decreases by adding 10% and 20% wood reinforcement. Reducing the amount of oxygen consumed when reinforced foams are burned is a safety benefit reducing the risk of asphyxiation. However, THR and TOC increased for foams with 30% reinforcement. Further investigations are needed to explain this result.

Table 4 presents the other parameters measured by cone calorimetry for RPUF foams. These results give an idea of the danger of intoxication posed by foam during a fire. The more the material gives off these gases, the greater the risk of suffocation and loss of consciousness. The values show that adding wood fiber in RPUF foams reduces this risk by reducing the amount of smoke and CO released for all formulations with 10 and 20% reinforcement. However, the CO_2_ content increases slightly for reinforced foams, probably due to the combustion of wood components. All reinforced foams show a decrease except PUR_01-30, with a slight increase in total smoke produced. The MAHRE provides information on the natural tendency of the material to develop a fire. The PUR_01 foams (fine granulometry) show an increase in this value depending on the percentage of reinforcement from 70 to 109 kW/m^2^, thus exceeding the foam without reinforcement (96.63 kW/m^2^). On the other hand, all other formulations have lower values than PUR0. Thus, adding wood reinforcement reduces the foam’s contribution to developing a fire [26]. One of the explanations for these results is that certain wood components such as lignin and cellulose degrade at higher temperatures under an oxidizing atmosphere. It has already been proven that lignin, for example, liquefies when subjected to high temperatures, thus forming a protective layer. In addition, wood could help form a layer of charcoal that protects the foam from the radiation produced by the fire. Another explanation for the improved fire retardancy of reinforced foams lies in the interaction of wood with isocyanate creating new urethane bonds. These slow down the flame spread during the decomposition of the foam with the release of non-combustible gases.

### 4.2. Effect of Mechanical Stirring on RPUFs Properties

#### 4.2.1. Density

The first effect of the mechanical stirring on the RPUFs properties was observed for the density. The increase in the density indicates a better dispersion of the wood reinforcement in the matrix. In the previous samples (manual stirring), wood flour residues were observed on the bottom layer of the samples. Such residues were absent when mechanical stirring was introduced. The change in the RPUF production process seems to overcome the difference in density between polyol and wood flour and promotes their dispersion in the matrix since all samples were taken out from the middle of the foam to ensure their homogeneity. The densities of the different formulations are shown in Figure 5. The results show proportionality between reinforcement’s percentage and foam density. Indeed, a higher reinforcement percentage leads to an increase in density. This is most likely due to a slower swelling rate of the foam, leading to denser samples. The higher density of the wood flour also contributes to this increase [27,28]. For example, foams reinforced with fine wood flour increased by 26% from 10 to 30 php. Moreover, the foams prepared with finer wood flour (<0.106 mm) have a higher density independently of the percentage. The foam density goes from 103 kg/m^3^ for PUM_01-30 to 81 kg/m^3^ for PUM_03-30. This decrease is related to the lower density of coarse wood particles and the higher specific surface for finer ones, which leads to a better reactivity between fiber and matrix, thus increasing the density. It is important to note that hydrogen and covalent bonds occur between wood flour, isocyanate, and polyol. Furthermore, the increase in density is more noticeable at 30 php. This is caused by the increase in viscosity when introducing a high percentage of reinforcement. In RPUFs foam, a high viscosity reduces the reaction’s kinetics and foam volume produced. Therefore, foams prepared with high reinforcement percentage have a higher mass and a lower volume causing an increase in density [17]. Besides, hydroxyl groups present in the wood flour act like nucleation sites which contributes to the formation of more cells, thus increasing the density of the foam [29,30]. This also explains the higher density for PUM_01. Indeed, fine wood flour is characterized by a higher specific surface. Therefore, it reacts better with isocyanate, producing a higher density [17]. These results are important since density correlates to the foam’s mechanical properties.

#### 4.2.2. Mechanical Properties

**Figure 6:**
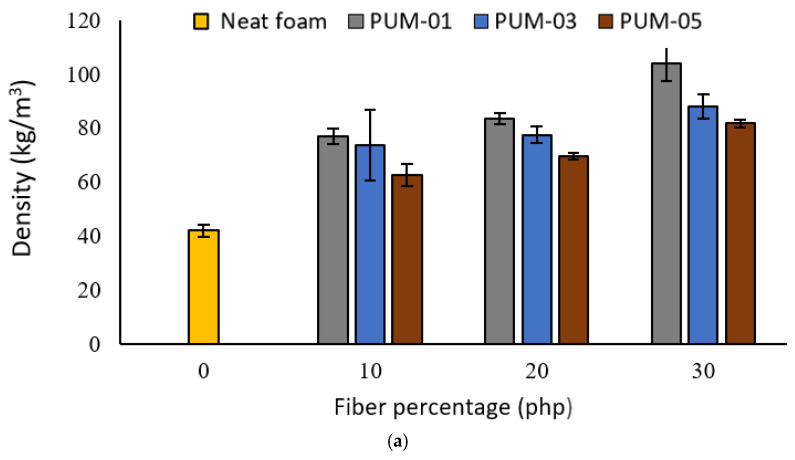

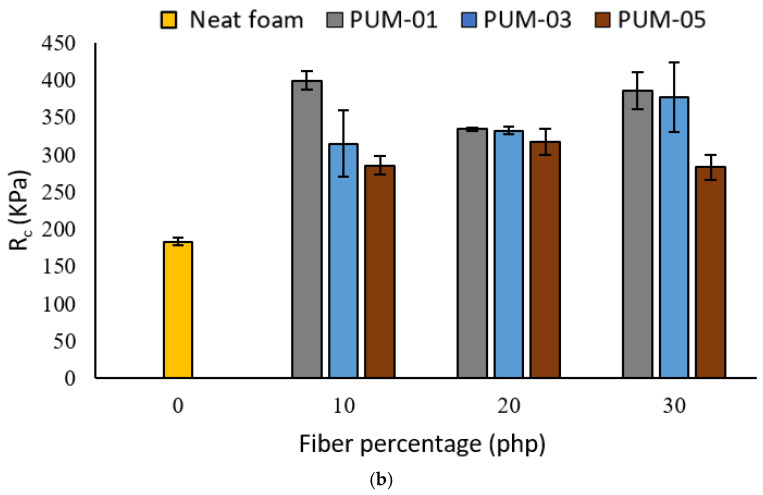
Effect of fiber particle size and percentage on reinforced foam properties: (**a**) density and (**b**) compressive strength.

One of the most important factors affecting a composite’s properties is homogeneity. This is the principal reason for which a mechanical stirring was adopted. The results of this process’s implementation on the foam’s mechanical properties are recorded in Figure 6.

The first observation was the overall increase in compression strength when mechanical stirring was compared to foams produced via manual stirring. Regardless of particle proportion and size, the mechanical mixing homogenizes the reactional mixture of polyol/wood and isocyanate, which procures a higher reinforcing effect of the wood flour. The maximum compressive strength for reinforced foams stirred manually went from 120 kPa for PU_01-10 to 400 kPa for PUM_01-10 (mechanical stirring). This increase was the result of two important factors. The first is the homogeneity of the polyol/wood flour mixture. Naturally, the increase in viscosity when wood flour is added to the polyol will decrease its reactivity, and wood particles will tend to agglomerate. The mechanical stirring provides a way to break those agglomerates and create well-dispersed particles in the matrix (Figure 6). Furthermore, a longer mixing time with a significantly higher speed will improve fiber wettability with polyol. This creates stronger covalent bonds between polyol and wood particles, leading to a higher reactivity when isocyanate is added to the mixture [13].

**Figure 7:**
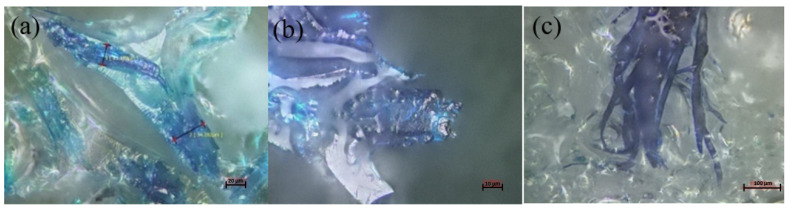
Microscopic observation of wood fiber-matrix interaction: (**a**,**b**) single fibers, (**c**) fiber agglomeration.

In this study, foams with smaller particle sizes had higher compressive strength regardless of the percentage added. The compressive strength also tends to increase proportionally to the percentage of wood particles added. This is most likely correlated to the increase in density seen in Figure 6 [31,32]. However, this was not the case for coarse particles, where foams prepared with 30 php showed a decrease in compressive strength. The nature of the reinforcement and its effect on the mixture’s viscosity are among the plausible explanations. The polyol/wood flour mixture for this particle size (>300 mm) tended to be a solid-like material, making it almost impossible to add isocyanate and react all the components with one another. This affected the foaming process and resulted in residual isocyanate that did not react with polyol or wood particles. Three theories could explain the effect of particle size on the reinforcement of foams. First, when the particle size is smaller than foam cell wall thickness, the particles become trapped inside the cell walls, struts, or junctions while reacting with isocyanate [33]. The second type of reinforcement possible is for medium size particles. Thus, fibers will entrap in the cavities of the cells where the insulating gas would be, creating a bridge between cell walls to reinforce the structure. In this reaction, fibers are not long enough to be hooked to both sides of the cell, and they will rather float in the cavity and do not contribute to the reinforcement of the foam. Finally, when long fibers are being used, the reinforcement mechanism is related to the intrinsic mechanical properties of the fiber itself. Furthermore, long fibers contribute more to stress transfer by providing more contact surfaces [34]. Furthermore, the fiber and the hydrogen bonds formed with isocyanate and covalent bonds between the fiber and polyurethane backbone provide even more reinforcement [35]. Figure 7 shows the different interactions between fiber and matrix. The observations for the different formulations were similar, with almost all the fibers coated with the matrix and most of them trapped in the cell walls. Nevertheless, we also observed some fiber agglomeration for higher reinforcement percentages, especially with longer, finer fibers.

In conclusion, the foams’ thin fibers with higher lengths provide better reinforcement properties. In our study, fiber length was similar for different particle sizes. However, the difference was in the fiber diameter, which explains why fiber with a size <0.106 mm produced the highest compressive strength. In addition, as mentioned before, fiber dispersion is as valuable as particle form and size since it prevents the aggregation of fibers which can be considered defects that reduce the foam strength. Hence, the importance of mechanical stirring when reinforcing foams with wood flour.

#### 4.2.3. Thermal Conductivity

For polyurethane foams, thermal conductivity is correlated to two main factors: cell morphology (open or closed cells, cell size, and distribution) and the blowing agent used. In this study, the blowing agent used is the same for all formulations. Thus, any change in thermal resistance is due to the morphology of the foams. This test was carried out for the foams with the best mechanical properties (PUM_01). The microscopy and morphology show that the percentage of reinforcement is the most influential factor in the size and shape of the reinforced foams.

Table 5 shows a slight variation in the thermal conductivity of the foams with the addition of reinforcement. Indeed, about an 8% increase is observed for the thermal conductivity of PUM_01-30 foam compared to that of PU0. Although there is no linear trend between the percentage of reinforcement with thermal conductivity, the incorporation of wood fiber reinforcement slightly reduces the insulating capacity of the foams. These results are not consistent with radical changes in foam morphology. Indeed, the irregular cell size and the presence of large microvoids in foams with 30% reinforcement should negatively affect thermal resistance [36]. On the other hand, the tests proved that the increase in thermal conductivity is not very important. Thus, the reinforcement has a negative effect on the insulating capacity of the foam, but this can only be confirmed by further studies addressing variations in the foam structure in terms of cell wall size and thickness.

In conclusion, the addition of wood reinforcement has a negative effect on the insulating properties of reinforced foams by modifying their structure. Nevertheless, the changes in thermal conductivity are not very significant. Optimizing the foam production process could induce a more homogeneous morphology with better cell size distribution and thus lead to better thermal resistance. This is consistent with the fact that commercially produced foams have much higher strengths than those prepared in the laboratory, largely due to the production process and cell size and shape [36].

### 4.3. Effect of the Fiber Treatment on RPUFs Properties

#### 4.3.1. Density

**Figure 8:**
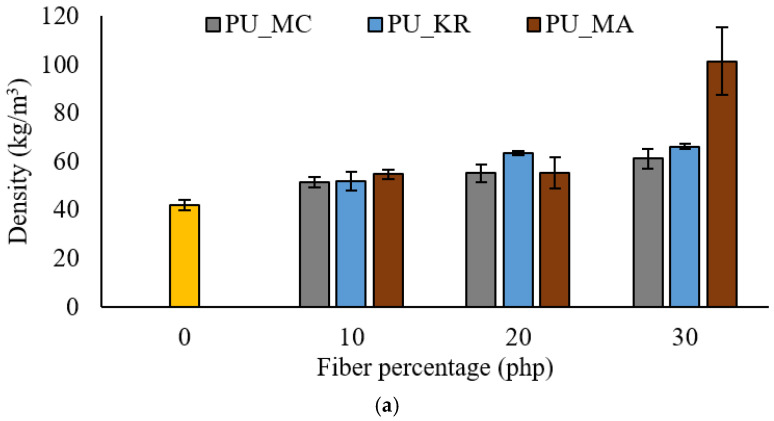

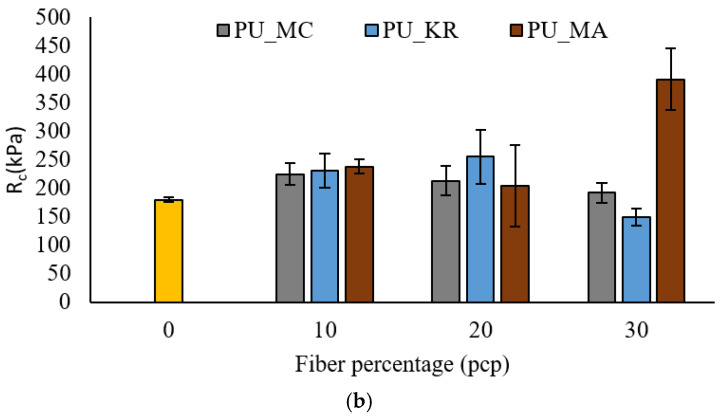
Effect of fiber treatment on reinforced foam properties: (**a**) density, (**b**) compressive strength.

**Figure 9:**
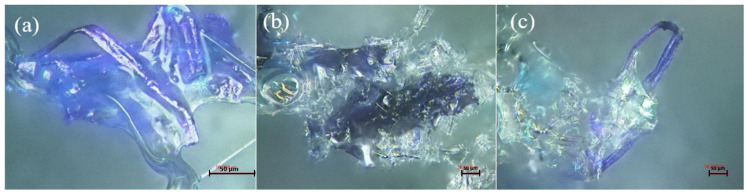
Microscopic observation of treated wood fibers in polyurethane matrix: (**a**,**b**) fiber adhesion, (**c**) non-reacted fiber.

The histogram (Figure 8) shows the densities of the foams reinforced with the treated fibers. Incorporating the treated fibers increased the density of all foams. This change in density is even more visible for PUM_MA-30 foam, going from 55.4 for 20 php reinforcement to 100 kg/m^3^ for 30 php. The low swelling at such a reinforcement proportion explains this result. At reinforcement rates of 10 and 20 php, foam with kraft fibers (PUM_KR) has the highest density. The type of fiber did not significantly affect the density, particularly at reinforcement rates of 10 and 20 php, due to the similarity between the used fibers. The comparison with the untreated reinforcement results, which showed a greater increase in density, could be explained because the treated fibers are more reactive with isocyanate, thus inhibiting polyol reactions with isocyanate. In addition, more hydroxyl bonds could contribute to the fiber agglomeration observed in Figure 9 [30]. Indeed, the gas releasing reactions granting the foam its final volume and the secondary reactions between isocyanate and various additives could have been disrupted by the excessive involvement of reinforcement with isocyanate. The higher hydroxyl group content of treated fibers is the principal cause of such involvement. A modification of the additives, particularly the catalyst, could compensate for this and allow the isocyanate to react first with the different main components of the foam and second with the reinforcements’ hydroxyl groups. Therefore, untreated fibers seem to be more suitable for the foam reinforcement used in this study. However, further studies considering additives, particularly surfactants and catalysts, could deepen the understanding of the observed phenomena and lead to more stable foams even with high hydroxyl index fibers [9,37].

#### 4.3.2. Mechanical Properties

Compressive strength at 10% deformation was evaluated for the treated fiber-reinforced foams (Figure 8). It can be seen here that the increase in strength compared to unreinforced foam (180 kPa) is less compared to that of untreated fiber-reinforced foams. This result corresponds to the changes in density, which provides the foam with most of its mechanical strength [38]. However, there is a fairly significant decrease for PUM_KR-30 to 149 kPa. The tendency of Kraft fibers to agglomerate creates inclusions in the foam structure and reduces its compression [39]. For PUM_MC foams, compressive strength decreases with an increasing percentage of reinforcement with a 15% reduction between PUM_MC-10 and PUM_MC-30, which is contradictory to density variation. The confocal microscopy results explain this phenomenon, showing the aggregation of such fibers in the cell struts and joints.

There is no trend linking the percentage of reinforcement and compressive strength (the standard deviation values are high). During the production of these foams, the tendency of the fibers to agglomerate induces foams with very variable densities, swellings, and resistances. Improving the fiber treatment process could reduce these variations in results and thus allow a better interpretation of their effect on polyurethane foams. However, the sudden increase in resistance for PUM_MA-30 is probably correlated with the increase in the density of this foam. Acetylated fibers have variable length and diameter and are significantly less reactive with polyol [14]. Foams produced with those fibers tend to have a very small volume compared to other fibers studied, thus producing very high-density foams. The results of the confocal microscopy (Figure 9) prove a good adhesion between the fiber and the matrix. The major problem observed for acetylated fibers was the high diameter; the fiber’s dimension interferes with the foam’s natural cell growth, thus hindering its mechanical properties. The smaller fiber kraft and MCC caused a lot of aggregation. Therefore, even with a great interaction with isocyanate, the fibers acted as a nucleation point and did not participate in the reticulation reactions, hence the average mechanical properties.

#### 4.3.3. Thermal Stability

**Figure 10:**
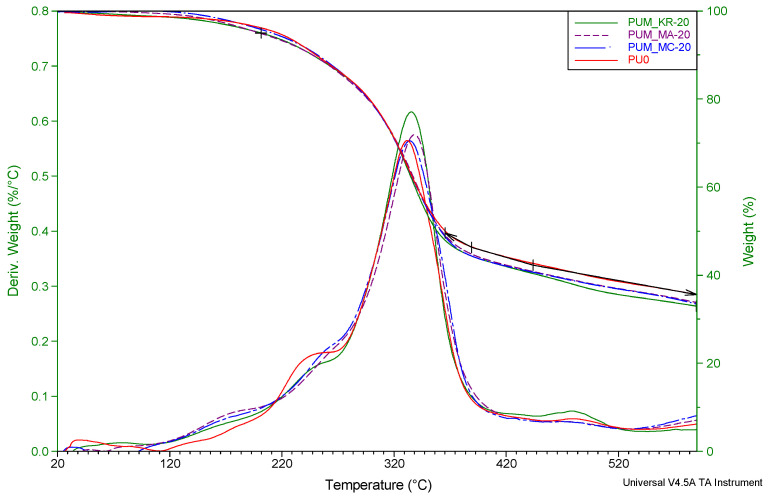
Effect of the different fiber treatments on TG and DTG of the RPUFs.

The DTG curves for PUM_MC/MA/KR-20 and PU0 (neat foam) are shown in Figure 10. As for untreated fiber-reinforced foams, the curve shows the beginning of degradation in the 100–200 °C range associated with the degradation of cellulose [23] contained in the fibers used as reinforcement. Two-phase degradation is still present for reinforced foams but is less pronounced, as shown by the DTG curves. The involvement of the various reinforcements in the production of urethane bonds makes them slightly more stable.

The different degradation temperatures show only a slight variation compared to the foam without reinforcement. Nevertheless, the maximum degradation temperature increased by incorporating treated fiber into the foam formulation. Temperatures at 5% and 50% mass loss have decreased, but the variations are not large enough to conclude that the thermal properties of the reinforced foams are deteriorating.

The introduction of chemically treated fibers slightly modifies the thermal properties reported by the ATG analysis. Still, these changes are not significant and do not induce a different thermal behavior from foam.

### 4.4. Effect of Wood Flour Reinforcement on Polyisocyanurate Foams

#### 4.4.1. Density

The reinforcing effect of untreated wood fibers that proved to produce the best results for polyurethane foams was introduced to polyisocyanurate (PIR) formulation. Foams with polyisocyanurate matrices are also used for thermal insulation. In these experiments, a percentage of 10 php will be used with the three granulometry because of this foam’s reactivity. Adding more than 10 php results in a mixture that is too viscous and thus causes the foam to collapse when it swells. Furthermore, a 10% addition of wood avoids fiber agglomeration. The main difference between the RPUF and PIR formulations is the reactivity of the reactional mix. For the PIR formula, the foams start rising after a 5-s window; this prevents the homogenization of the polyol, fiber, and isocyanate mixture.

**Figure 11:**
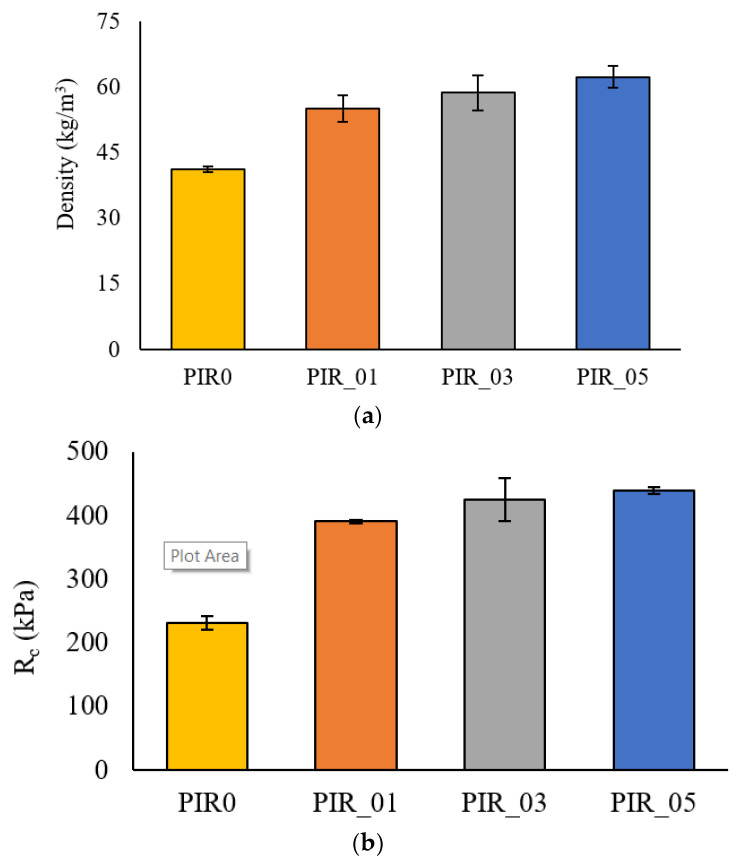
Effect of particle size on reinforced Polyisocyanurate foam properties: (**a**) density, (**b**) compressive strength.

The effect of the untreated fiber reinforcement diameter on the density of the individual PIR foams is reported in Figure 11. An addition of 10% reinforcement shows a significant increase in density. The most significant increase is recorded for PIR_05-10, with a more than 50% increase from 41 kg/m^3^ for PIR0 to 62 kg/m^3^. These results are expected given the variations observed with RPUF foams. The viscosity of the polyol/reinforcement mixture is the main reason for density changes by partially inhibiting foam swelling. In addition, the wood fiber reinforcement has a higher density than polyisocyanurate, which leads to an overall increase in the density of the composite. Despite the speed and reactivity of PIR compared to polyurethane foams, the addition of wood fiber reinforcement induces the same behavior at the density level. The density increases with the increase in the reinforcement size. These results differ from RPUF foams mainly because of the morphology of PIR foams, which generally have smaller cell sizes. For PIR foam, it is recommended to use a catalyst to promote crosslinking to create isocyanurate bonds. In addition, the formulation of PIR foams contains a significant excess of isocyanate.

#### 4.4.2. Mechanical Properties

Figure 11 shows the compressive strengths at 10% deformation for PIR foams with and without reinforcement. The changes made to PIR foams due to the addition of untreated wood reinforcements are similar to RPUF foam. The compressive strength has increased from 230 kPa for PIR0 to 439 kPa for PIR_05-10. This increase is tightly correlated to the variations in foam density which grants a denser foam, thus increasing its compressive strength [31].

However, there is a difference in the behavior compared to RPUF foams, where increasing the size of the reinforcement decreased the mechanical properties of the 10 php reinforcement foam. Indeed, we noticed a slight increase in Rc for coarse reinforcements of PIR foam compared to medium-sized reinforcements. However, this variation is not significant. PIR and RPUF foams are chemically similar but morphologically and kinetically very different [40].

**Figure 12:**
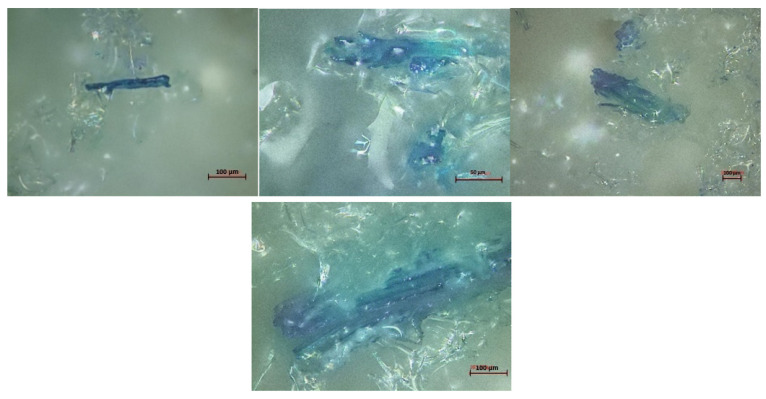
Microscopic observation of single fibers interaction with Polyisocyanurate foam.

Indeed, PIR foam is much more reactive, with a mixing time between isocyanate and polyol of 5 s before the foam starts to swell. The addition of reinforcement only slightly affected the reaction kinetics of the foam, unlike RPUF. This could explain the absence of the effect of reinforcement size on mechanical properties. Indeed, the isocyanate will naturally react with the hydroxyl groups present in the polyol as a priority and then react with those present in the wood reinforcement. The speed of foam swelling does not allow enough time for the wood to influence the natural kinetics of the foam, and thus the effect of the size of the reinforcement will be less visible. In addition, the cell sizes for PIR foams are much smaller than the RPUFs. Therefore, even the finer fibers cannot be trapped in the cell walls to enhance the mechanical properties of the foam-like its shown in Figure 12. The fibers react with the foam but disrupt its cell structure.

#### 4.4.3. Thermal Conductivity

The results showed that the thermal conductivity of PIR foams is slightly lower than that of RPUF foams. This can be explained by the cells’ small size and larger number and the rapid solidification of the cell walls allowing the imprisonment of a greater quantity of gas in the cells [36].

**Figure 13:**
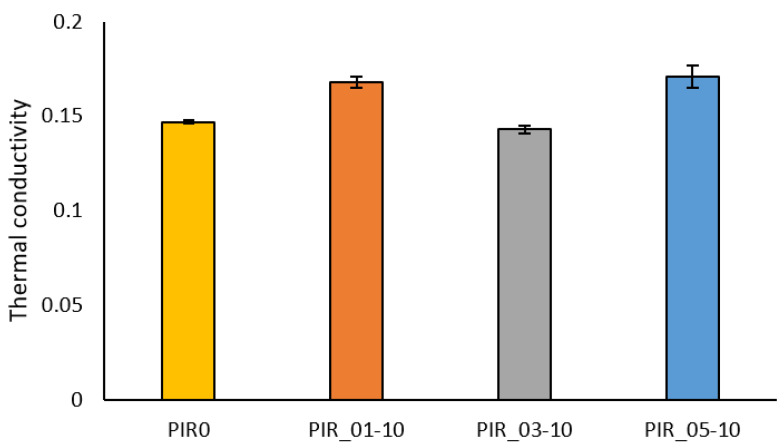
Effect of particle size on the thermal conductivity of reinforced Polyisocyanurate foams.

The effect of particle size on the different formulations’ thermal conductivity was summarized in Figure 13. PIR_01/05-10 foams have a 16% increase in thermal conductivity from 0.147 W/m·K for PIR0 to 0.171 W/m·K for PIR_05-10. For foams with a medium reinforcement size, the conductivity has decreased slightly. At 10 php reinforcement, the change in conductivity does not significantly affect the insulating power of the foam. However, a change in the formulation of the PIR foam could allow a higher percentage of reinforcement. The percentage of open cells in the foam confirms that the addition of lignocellulosic fibers reinforces the PIR foams.

## 5. Conclusions

Rigid polyurethane foams with different filler types and sizes were successfully produced. The fillers used confirmed the interaction between wood fibers and isocyanate and their adhesion to the matrix. Coarse particles were not suitable for the reinforcement of RPUFs. Furthermore, the mechanical steering improved the compressive strength compared to manual stirring. The fiber treatments did not improve the foam’s mechanical properties, mainly caused by the aggregation of treated fibers, thus creating irregularities in the three-dimensional structure of the foam. At 10 php, adding wood fibers improved the compressive strength of both polyurethane and polyisocyanurate foams. Wood fibers slightly improved the foams’ thermal stability, thermal conductivity, and fire retardancy.

## Figures and Tables

**Table 1:** Polyurethane foam formulation.

	Composition	Percentage (%)
Component A (Polyol+additifs)	Polyol	50–70
	Diméthylclohexylamine	0.5–2
	Hydrofluorocarbures	15–30
	Fire retardant	15–20
Component B (Isocyanate)	Mixture of MDI	100

**Table 2:** Polyisocyanurate foam formulation.

Raw Material	Composition	Parts per Hundred Polyols (php)
polyol	-	100
Isocyanate	MDI	Poly/iso ratio 60/100
Fibers	Black Spruce (3 particle sizes)	10

**Table 3:** Nomenclature for RPUF and PIR reinforced foams.

Effect of Particle Size		Effect of Type Treatment	
PUR/PIR_X	Size	PU/PIR_Y	Fiber Type
01	<0.106 mm	MA	Acetylated fibers
03	0.1–0.3 mm	KR	Kraft fibers
05	0.3–0.5 mm	MC	Microcrystalline cellulose

**Table 4:** Fire retardancy properties of RPUF foams as a function of grain size and reinforcement proportion.

	Properties			
Foam	TSR(m^2^/m^2^)	MCOY (kg/kg)	MCO_2_Y (kg/kg)	MAHRE (kW/m^2^)
Reference	120.84 ± 35.42	0.085 ± 0.000	1.05 ± 0.07	96.63 ± 2.86
PUR_01-10	62.87 ± 19.08	0.078 ± 0.016	1.24 ± 0.10	70.73 ± 7.70
PUR_01-20	95.72 ± 25.27	0.078 ± 0.01	1.20 ± 0.096	90.57 ± 12.82
PUR_01-30	127.3 ± 28.6	0.088 ± 0.007	1.15 ± 0.003	109.88 ± 10.18
PUR_03-10	91.59 ± 7.85	0.079 ± 0.001	1.10 ± 0.07	86.12 ± 3.74
PUR_03-20	65.95	0.064	1.14	84.41
PUR_03-30	100.18 ± 10.04	0.072 ± 0.006	1.14 ± 0.05	87.50 ± 3.86
PUR_05-10	67.53 ± 3.45	0.065 ± 0.006	1.06 ± 0.08	81.52 ± 2.92
PUR_05-20	66.82 ± 9.96	0.067 ± 0.007	1.12 ± 0.13	74.92 ± 2.41
PUR_05-30	105.72 ± 18.88	0.075 ± 0.004	1.15 ± 0.08	93.29 ± 7.80

The total smoke released (TSR), Mean CO yield (MCOY), Mean CO_2_ yield (MCO_2_Y), the maximum average rate of heat emission (MARHE).

**Table 5:** Effect of fiber percentage on reinforced Polyurethane foam thermal conductivity.

Formulation	Thermal Conductivity, (W/m·K)
PU0	0.155 ± 0.02
PUM_01-10	0.164 ± 0.001
PUM_01-20	0.159 ± 0.012
PUM_01-30	0.167 ± 0.005

## Data Availability

Available data can be found in the student thesis at https://depositum.uqat.ca/id/eprint/836 accessed on 27 April 2022.

## Supplementary Materials

The following supporting information can be downloaded at: https://www.mdpi.com/article/10.3390/ma15124316/s1. Figure S1. Iso-curves for compressive strength. Table S1. Effect of particle size and percentage on compressive strength.
