# Supplementary material for: Synthesis and Characterization of Wood Rigid Polyurethane Composites
Source: Materials (Basel). 2022 Jun 18;15(12):4316. doi: 10.3390/ma15124316 (PMC9229658; doi:10.3390/ma15124316)
Supplement: Supplementary file 1 [file materials-15-04316-s001.zip › materials-1724833-supplementary.pdf]

Supplementary Material

Synthesis and Characterization of Wood Rigid Polyurethane Composites

Hamza Bradai [1], Ahmed Koubaa [1*], Hassine Bouafif [2], Armand Langlois [3] and Basma Samet [4]

[1] Institut de Recherche sur Les Forêts, Université du Québec en Abitibi-Témiscamingue, 445 Boul. Université, Rouyn-Noranda, QC J9X 5E4, Canada; hamza.bradai@uqat.ca (H.Br.)
[2] Centre technologique des résidus industriels (CTRI), Rouyn-Noranda, QC J9X 5E5, Canada; hassine.bouafif@cegepat.qc.ca
[3] EnerLab, 1895 Chemin de l'Industrie, Saint-Mathieu-de-Beloeil, QC J3G 4S5, Canada, alanglois@enerlab.ca
[4] National Engineering School of Sfax (ENIS), University of Sfax, Sfax 3038, Tunisia; Basma.samet@enis.tn
* Correspondence: ahmed.koubaa@uqat.ca

Experimental plan and statistical analysis

A composite experimental plan determined the optimal formulations in density and mechanical resistance using the NEMROD statistical analysis [1]. The iso-response surfaces of density and mechanical properties gave a simple visualization of the optimum fiber size and proportion.

The NEMROD statistical analysis identified foam formulations' optimum percentage and fiber size. The effect of these factors was not significant on the foam density. On the other hand, the effect of the particle size and its interaction with percentage was significant for the compressive strength (Table S1).

Table S1. Effect of particle size and percentage on compressive strength.

Coeficient	Value	Pr(>F)
b1	-2,917	16,9
b2	-8,833	0,142 **
b1-1	-2,250	52,2
b2-2	-23,000	< 0,01 ***
b1-2	9,125	0,408 **

R^2=0,69, '***' 0,001 '**' 0,01 '*' 0,05.

The iso-response (Figure S1) shows that the optimal compressive resistance is obtained with a particle size between 250 and 125 μm and percentages under 15%. It is known that the compressive strength for foams used in construction needs to be at least 100 kPa. Thus, the particle size cannot be higher than 300 μm, with percentages going from 10 to 30 %. The crosshatched area gives an idea of the acceptable percentage and particle size, while the colored area represents the maximum compressive strength achievable.

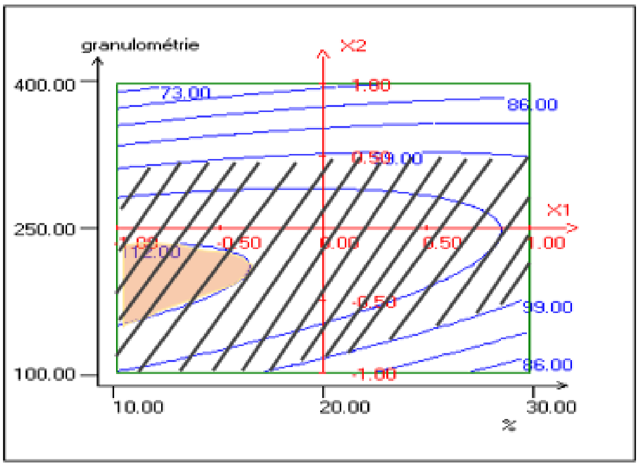

Figure S1. Iso-curves for compressive strength.

References

1. Tinsson, W. *Plans d'Expérience: Constructions et Analyses Statistiques*, 1st ed; Mathématiques et Applications; Springer: Berlin/Heidelberg, Germany, 2010; Volume 67. https://doi.org/10.1007/978-3-642-11472-4
